# Multi-Modality Imaging Approach in a Challenging Case of Surgically Corrected Partial Anomalous Pulmonary Venous Return and Atrial Tachycardia Treated With Radiofrequency Ablation

**DOI:** 10.7759/cureus.13009

**Published:** 2021-01-30

**Authors:** Emanuele Muscogiuri, Marco Di Girolamo, Carmen Adduci, Pietro Francia, Andrea Laghi

**Affiliations:** 1 Radiology, Sant'Andrea Hospital - Sapienza University of Rome, Roma, ITA; 2 Cardiology, Sant'Andrea Hospital - Sapienza University of Rome, Roma, ITA

**Keywords:** partial anomalous venous return, cardiac magnetic resonance, cardiac computed tomography, transthoracic echocardiography, cardiac surgery

## Abstract

Pulmonary anomalous venous return (PAPVR) is defined as a congenital anomaly in which at least one but not all of the pulmonary veins abnormally drain into a systemic vein or directly into the right atrium. Signs and symptoms related to this condition are due to the hemodynamic abnormalities secondary to left-to-right shunt and the possible presence of other associated cardiac anomalies (e.g., sinus venous atrial septal defect). Therefore, depending on the extent of the shunt, the clinical presentation of PAPVR is variable, ranging from asymptomatic patients to patients affected by severe heart failure with right-sided volume overload. PAPVR with a clinically significant shunt should be referred for surgical correction with different techniques depending on the presence of associated cardiac anomalies.

We are presenting a case of partial anomalous venous return (PAPVR) in a 66-year-old man who underwent surgery 26 years ago to correct an anomalous venous connection between the right superior pulmonary vein (RSPV) and the superior vena cava (SVC) through a veno-atrial baffle. The patient was admitted to the emergency department due to atrial tachycardia. Trans-thoracic echocardiography (TTE) showed a dilated right ventricle (RV) with mild RV systolic dysfunction and pulmonary hypertension. Cardiac magnetic resonance (CMR) further confirmed the findings described by TTE and also demonstrated areas of fibrosis replacement in the hinge points. Cardiac computed tomography (CCT) was able to accurately depict and evaluate the surgically created veno-atrial baffle and also showed an anomalous connection between the left superior pulmonary vein (LSPV) and the brachiocephalic vein (BCV) through a vertical vein. The patient was successfully treated with radiofrequency ablation for his arrhythmia.

## Introduction

Pulmonary anomalous venous return (PAPVR) is a congenital anomaly in which at least one but not all pulmonary veins abnormally drain into a systemic vein or directly into right atrium [1]. PAPVR is a congenital anomaly involving the development of the pulmonary veins. Instead of connecting with the left atrium, in this condition the pulmonary vein may connect directly into: 1) the right atrium; 2) derivatives of the right common cardinal system (i.e., superior vena cava and azygos vein); 3) derivatives of the left common cardinal system (i.e., the coronary sinus); 4) the umbilicovitelline system through the portal vein and venous duct [2]. Depending on the site of the anomalous connection, PAPVRs can be categorized as supra-cardiac (e.g., connection to the superior vena cava), cardiac (e.g., connection to the right atrium), infra-cardiac or infra-diaphragmatic (e.g., connection to inferior vena cava) and mixed types [3]. Depending on the number of the pulmonary veins involved by the malformation, PAPVRs can further also be classified as a single-branch type, unilateral two-branch type and bilateral single-branch type [4].

The clinical presentation of this condition is strictly dependent on the entity of the left-to-right shunt due to the anomalous pulmonary flow return and possible associated cardiac malformations (e.g., sinus venosus atrial septal defect being the most common). In cases in which the shunt is not clinically significant (Qp/Qs<50%), the patients can be asymptomatic until adulthood [5, 6]. Conversely, patients with a clinically significant shunt show symptoms including decreased functional capacity, exertional shortness of breath and less frequently pulmonary infections and signs of right heart failure. Pulmonary artery pressures can be normal but generally increase with age [5]. These findings are associated with chronic right-sided volume overload, leading to pulmonary overcirculation and ultimately heart failure [3, 5]. Increased pulmonary vascular resistance and reversal of left-to-right shunt with the development of an Eisenmenger syndrome is also an infrequent possibility [3, 7].

Cases of PAPVRs with a clinically significant left-to right shunt (Qp/Qs>1.5, demonstrated by pulmonary angiography or magnetic resonance imaging) should be referred for surgical correction. Patients who developed pulmonary arterial hypertension (PAH) should be carefully evaluated prior to surgery since the correction might prove ineffective in this peculiar class of patients and it could be even harmful for patients untreated for PAH due to the risk of acute right heart failure following the procedure [8].

## Case presentation

A 66-year-old man was admitted to our Emergency Department complaining of palpitations. Twenty-six years previously, the patient had undergone surgery for partial anomalous venous return (PAPVR). The operative report showed the patient had an anomalous venous connection between the right superior pulmonary vein (RSPV) and the superior vena cava (SVC) 3 cm from the cavo-atrial junction, with right atrial (RA) and right ventricular (RV) dilation. No further anomalies were described regarding pulmonary venous drainage or other congenital malformations responsible for left-to-right shunt. PAPVR was corrected by creating a veno-atrial baffle connecting RSPV to left atrium (LA). There was no mention regarding the surgery on the SVC.

**Figure 1:**
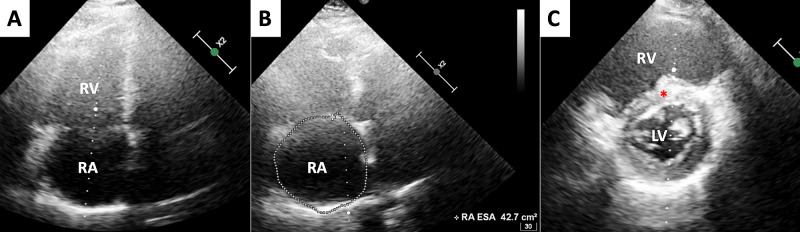
Transthoracic echocardiography: Functional and morphological evaluation (A,B) Four Chambers view, end-diastolic phase. The images show dilated right atrium (42.7 cm^2^ area) and right ventricle (RV). (C) Short axis view. The image confirms RV dilation with a mild interventricular septal flattening (red asterisk) and a D-shaped appearance of the left ventricle.

On admission, the electrocardiogram (ECG) showed atrial tachycardia (AT, cycle length: 250 ms) with a heart rate of 120 bpm. Transthoracic echocardiography (TTE) showed RV and RA enlargement, mild RV systolic dysfunction (approximately 45-50% ejection fraction with tricuspid annular plane excursion of 16 mm) [9], mild pulmonary arterial hypertension with systolic pulmonary pressure (PAP) of 40 mmHg, and D-shaped left ventricle, consistent with increased RV filling pressure (Figure 1). Qp/Qs estimated by TTE was equal to 1.5.

After unsuccessful pharmacological cardioversion with amiodarone, electrical cardioversion was attempted. Transient sinus bradycardia was achieved but was followed by early arrhythmia relapse. Therefore, an electrophysiologic (EP) study and catheter ablation of the arrhythmia was planned.

Cardiac magnetic resonance (CMR) and cardiac computed tomography (CCT) were performed before ablation since the patient did not have any previous imaging records.

At first, the patient underwent CMR. Cine balanced-steady state free precession (b-SSFP) sequences were performed on multiple planes in order to evaluate heart volumes, function and anatomical features. Phase-contrast (PC) sequences were performed on the ascending aorta and main pulmonary artery in order to estimate a left-to-right shunt. Phase-sensitive inversion recovery (PSIR) sequences were performed to detect areas of late gadolinium enhancement (LGE).

**Figure 2:**
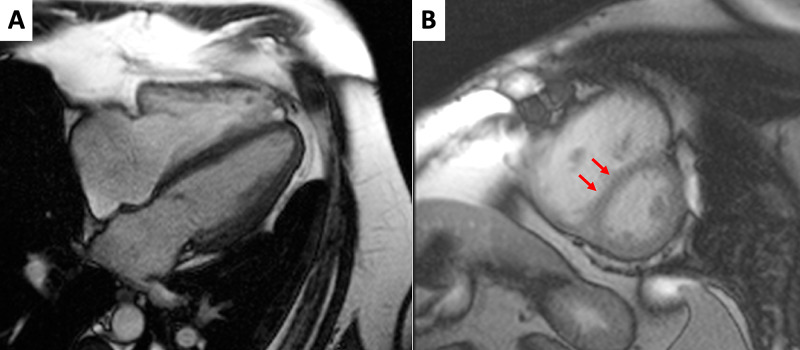
Cardiac magnetic resonance: morphology and function evaluation (A) Pre-contrast balanced Steady State Free Precession (b-SSFP) sequences, Horizontal Long Axis plane, end-diastolic phase. The right atrium and ventricle (RV) appear mildly dilated, while the left atrium and ventricle are morphologically normal. (B) post-contrast b-SSFP sequences, Short Axis plane, midventricular segment, end-diastolic phase. RV appears dilated and hypertrophic; furthermore, inter-ventricular septum is mildly flattened (arrows).

**Figure 3:**
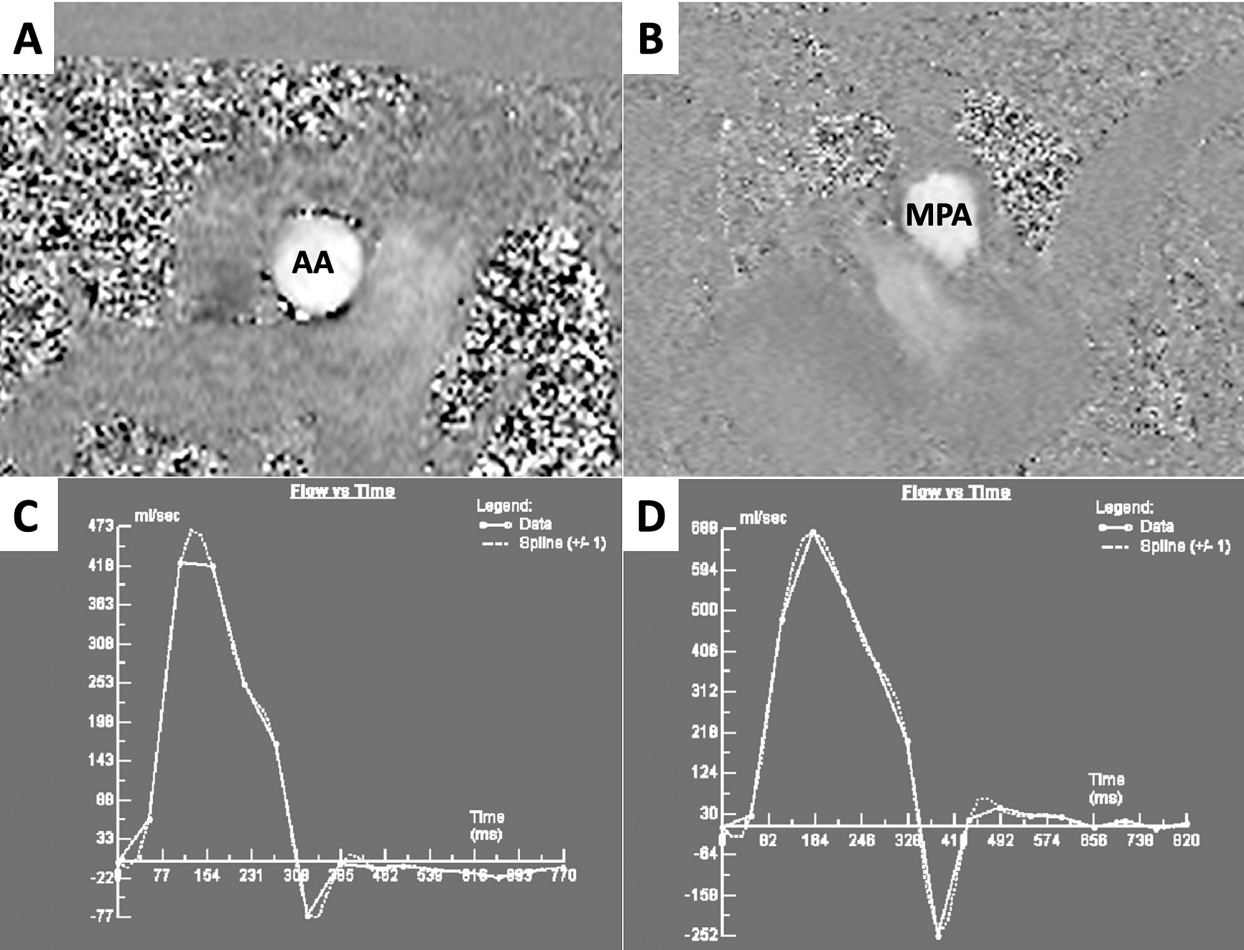
Cardiac magnetic resonance: flow measurements and time-to-flow curves (A) Phase contrast sequences (PC) showing blood flow through the ascending aorta (AO) in meso-systolic phase. (B) PC showing blood flow through the main pulmonary artery (MPA), in meso-systolic phase. (C) Time-to-flow curve obtained by post-processing of PC images in (A). Net stroke volume through AO was equal to 64 ml. (D) Time-to-flow curve obtained by post-processing of PC images in (B). Net stroke volume through MPA was equal to 119 ml.

**Figure 4:**
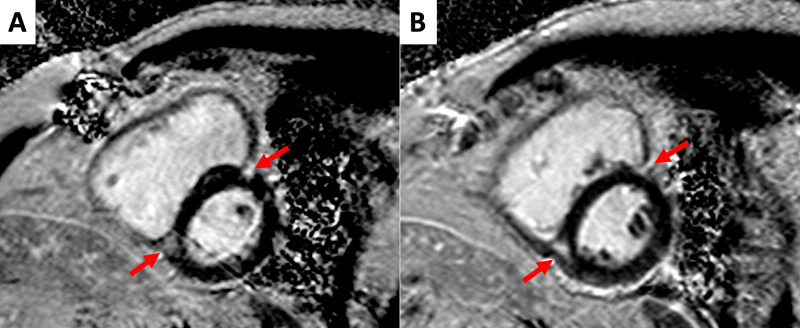
Cardiac magnetic resonance: tissue characterization (A) Phase sensitive inversion recovery sequences (PSIR), short axis plane, basal segments. (B) PSIR, short axis plane, midventricular segments. In both images it is possible to notice late gadolinium enhancement (LGE) areas in the right ventricle insertion points (arrows).

Cine b-SSFP sequences showed mild right ventricle dilation (End diastolic indexed volume: 126 ml/m^2^) and systolic dysfunction (Ejection fraction: 47%). Left ventricle (LV) volumes and function were in normal ranges, although a diastolic flattening of inter-ventricular septum was observed, most likely due to increased right ventricle filling pressure (Figure 2). Following post-processing of PC sequences’ images, CMR exam confirmed left-to-right shunt, with a Qp/Qs: 1.86 (Figure 3). PSIR sequences showed LGE areas in RV insertion points, likely due to focal fibrotic replacement (Figure 4).

However, CMR was not of help regarding anatomical evaluation. Due to difficulty in breath-hold sequences, we were not able to properly visualize the baffle and pulmonary veins.

Subsequently, the patient underwent CCT performed with a 256-detector rows scanner (Philips iCT 256, Philips, The Netherlands) with a retrospective ECG-gated acquisition protocol. The scans were performed during intravenous injection of iodinated contrast medium (70 ml of Iomeprol, 400 mgI/ml Iomeron, Bracco Imaging, Italy) with a flow rate of 5 ml/s and a saline bolus chaser of 40 ml. Images were further reconstructed using a dedicated workstation and performing 3D reconstructions.

**Figure 5:**
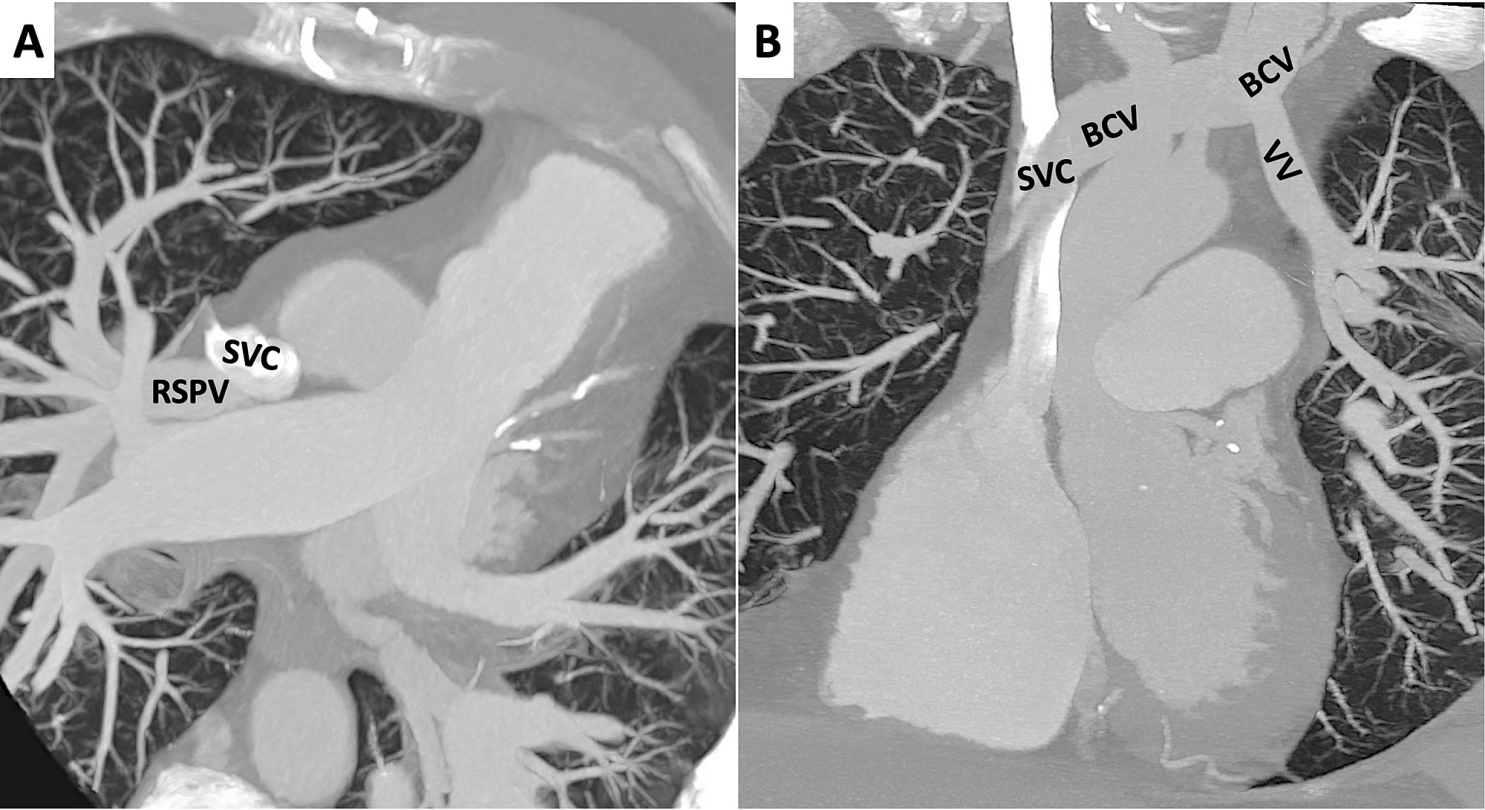
Cardiac computed tomography: Anomalous venous return (A) Maximum Intensity Projection (MIP) images in axial plane. Right superior pulmonary vein (RSPV) drains into the superior vena cava (SVC). (B) MIP images in coronal plane. Left superior pulmonary vein drains into the brachiocephalic vein (BCV) through a vertical vein (VV) and the latter drains into the SVC.

**Figure 6:**
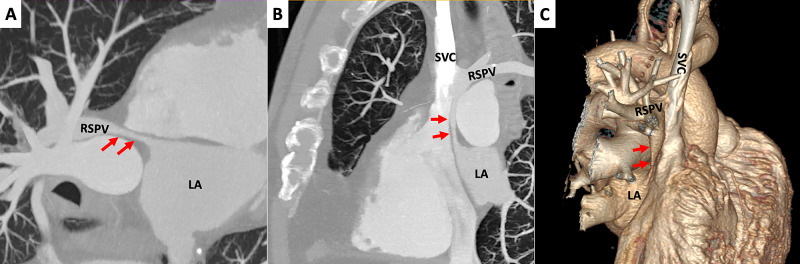
Cardiac computed tomography: surgically created baffle (A,B) Maximum intensity projection (MIP) images in oblique plane and coronal plane, respectively. It is possible to see the baffle (arrows) connecting the right superior pulmonary vein (RSPV) to the roof of left atrium (LA). Furthermore, in (B), we can also see again RSPV draining into superior vena cava (SVC). (C) Volume rendering images. In this 3D image it is possible to evaluate the course of the baffle, from inferior aspect of RSPV to the roof of LA.

**Figure 7:**
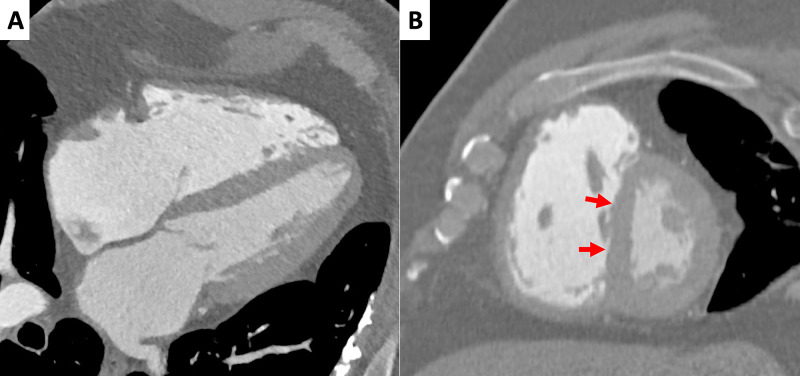
Cardiac computed tomography: functional and morphological evaluation (A,B) Horizontal long axis and short axis plane, respectively; 80% R-R interval. The images confirm right atrium and ventricle dilation, with hypertrophic appearance of the latter, as seen before in cardiac magnetic resonance scans. Furthermore, the left ventricle appears «D-shaped» due to interventricular septum flattening (arrows).

CCT scan confirmed anomalous drainage of RSPV in SVC. We observed the left superior pulmonary vein (LSPV) draining into the brachiocephalic vein (BCV) through a vertical vein (Figure 5). The baffle was visualized (3 mm diameter in the narrowest point) connecting the RSPV to the LA roof (Figure 6). Indirect signs of pulmonary hypertension were also present, including increased diameters of the right and left pulmonary arteries (28 mm and 30 mm, respectively) and a segmental artery-to-bronchial diameter ratio greater than 1:1. We also observed interventricular septal diastolic flattening, with a D-shaped morphology of LV in short axis plane reconstructions at 80% R-R interval, confirming the finding we described before regarding CMR scan (Figure 7). No atrial septum defects (ASD) were detected by any imaging technique used. No significant residual lung parenchymal abnormalities associated with PAPVR were detected. Incidentally noted was some residual pleural thickening and calcification on the left side.

Following CCT scan, the patient underwent an ablation procedure. 3D electroanatomic mapping (EnSite NavX, Abbott) during tachycardia revealed a centrifugal atrial activation pattern with earliest, low amplitude and fractionated electrograms on the interatrial septum, at the level of the inferior border of the baffle’s ostium. Pacing manoeuvres were consistent with micro-reentrant atrial tachycardia. Radiofrequency was delivered at the target zone with immediate termination of the tachycardia. A cluster of consolidating ablation lesions were delivered at the location. No further atrial tachycardia was induced with burst pacing after ablation. No arrhythmia recurrences were observed during a four-month follow-up.

## Discussion

We present a case of supra-cardiac bilateral single-branch PAPVR with RSPV and LSPV anomalous drainage in SVC and BCV, respectively. Right-sided anomalous connections are the most common (90% of PAPVR) and often associated with sinus venosus type atrial septal defects (80% of cases), while cases like ours, characterized by intact interatrial septum represent approximately 3% of cases [10]. The condition described is very rare among general population. A retrospective study by Ho et al. found just two cases of bilateral upper lobes PAPVR out of 45,538 contrast-enhanced chest CT scans performed during eight years [11].

Regarding the clinical features of our case, AT might have been the consequence of the surgical procedure the patient underwent previously. This surgical technique, as also other techniques performed to correct other congenital conditions (e.g., Fontan procedure, atrial septal defects correction, etc.) may be associated with the late occurrence of atrial arrhythmias [10,12].

As expected, our patient showed signs of PAH secondary to a clinically significant left-to-right shunt, as shown by TTE, CMR and CT.

TTE is the first-line diagnostic technique in these patients according to the latest European Society of Cardiology (ESC) guidelines [5]. This technique was able to provide an accurate outlook about RV function, detecting signs of right-sided volume overload and systolic dysfunction and estimating PAP. However, TTE did not allow to properly evaluate neither the veno-atrial baffle nor the anomalous connections of the pulmonary veins.

CMR proved useful not only in confirming the findings showed by TTE, precisely calculating the Qp/Qs, but also for performing myocardial characterization. The Qp/Qs ratio is considered to be more accurate than that calculated by TTE, since it is well established that CMR is able to provide a more precise quantification. This is especially useful in the preoperative evaluation of patients affected by PAPVR. CMR images also demonstrated areas of fibrotic replacement in the RV insertion points, likely due to chronic volume overload, but the prognostic value of this finding is still uncertain [12]. Beside heart morpho-functional evaluation and left-to-right shunt quantification, it was important to visualize the veno-atrial baffle, since baffle obstruction could be a potential complication of the surgery the patient had undergone [13]. CMR is the best imaging techniques of choice in pulmonary vessels anatomical assessment and it can be used to properly evaluate baffle lumen and course with a dedicated protocol [14]. However, patients who do not cooperate during breath-hold sequences may potentially impair the image quality, thus making difficult to achieve optimal anatomical definition.

Finally, CCT allowed to detect the LPSV draining to BCV, that was unknown at first, also precisely depicting the veno-atrial baffle course and patency. Moreover, this technique also allowed to reconstruct 3D volume rendering images, further helping to understand thoroughly the anatomy of the patient.

## Conclusions

Our case highlights the way that TTE, CMR and CCT proved useful in the preoperative evaluation of a patient affected by a rare type of PAPVR, each study providing different and valuable information. In patients with grown-up congenital heart (GUCH), this provides proper evaluation of the surgical procedure and any late complications.
